# Tuning of the Mg Alloy AZ31 Anodizing Process for Biodegradable Implants

**DOI:** 10.1021/acsami.0c22933

**Published:** 2021-03-11

**Authors:** Andrea Zaffora, Francesco Di Franco, Danilo Virtù, Francesco Carfì Pavia, Giulio Ghersi, Sannakaisa Virtanen, Monica Santamaria

**Affiliations:** † Dipartimento di Ingegneria, Università degli Studi di Palermo, Viale delle Scienze, Palermo 90128, Italy; ‡ Dipartimento di Scienze e Tecnologie Biologiche, Università degli Studi di Palermo, Chimiche e Farmaceutiche (STEBICEF), Viale delle Scienze, Palermo 90128, Italy; § Chair for Surface Science and Corrosion, Department of Materials Science and Engineering, University of Erlangen-Nürnberg, Erlangen 91058, Germany

**Keywords:** biomedical, corrosion resistance, electrochemical impedance spectroscopy, hard anodizing, Mg alloy

## Abstract

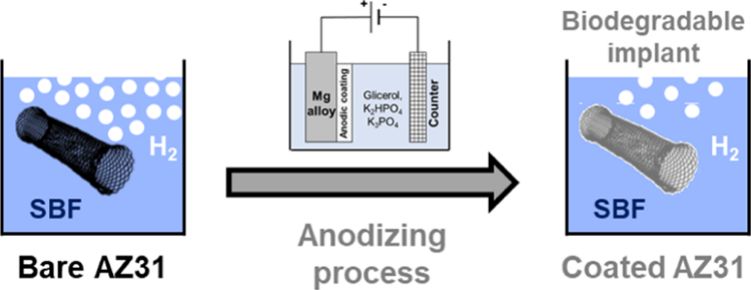

Coatings were grown on the AZ31 Mg alloy by a hard anodizing process in the hot glycerol phosphate-containing electrolyte. Anodizing conditions were optimized, maximizing corrosion resistance estimated by impedance measurements carried out in Hank’s solution at 37 °C. A post anodizing annealing treatment (350 °C for 24 h) allowed us to further enhance the corrosion resistance of the coatings mainly containing magnesium phosphate according to energy-dispersive X-ray spectroscopy and Raman analyses. Gravimetric measurements revealed a hydrogen evolution rate within the limits acceptable for application of AZ31 in biomedical devices. *In vitro* tests demonstrated that the coatings are biocompatible with a preosteoblast cell line.

## 1 Introduction

Metallic biomaterials are nowadays widely used in human body as implants (e.g., artificial joints, stents, and bone plates). However, serious concerns could arise using permanent metallic implants since they could cause harmful body reactions (e.g., thrombosis, physical irritation, restenosis, etc.) and also an inability to adapt to the growth and changes in the human body.^1,2^ In these cases, additional surgery is needed to remove metallic implants but only after the tissue healing function has been completed. Biodegradable implants can represent a solution because they can be implanted for an appropriate period to fix and then disappear, avoiding undesirable body reactions.^3,4^ Magnesium alloys have attracted increasing attention over the last two decades, of both industrial and academic world, as a suitable candidate for biodegradable biomedical devices (e.g., vascular stents and bone plates)^5,6^ due to their peculiar properties, such as high strength/weight ratio, good thermal and electrical conductivities, excellent vibration, and shock absorption.^7^ Indeed, with respect to materials such as stainless steel, titanium alloys, and Cr–Co based alloys, which are typically employed, Mg and its alloys have a low density (1.74–2.0 g cm^–3^) and an elastic modulus between 41 and 45 GPa, and once implanted in the human body (*in vivo*), the ions and/or particles released as a consequence of corrosion phenomena are not detrimental to the body.^8^

However, the potential clinical applications of Mg alloys have been hindered by their poor corrosion resistance. Due to the very negative reduction potential of Mg and its poor corrosion resistance in chloride-containing environments (human body fluids or blood plasma), the degradation rate of magnesium and its alloys is so high that the mechanical integrity before the diseased or damaged bone tissue healed is not always maintained. Moreover, since water reduction is the common cathodic process during corrosion of Mg and its alloys, the high corrosion rate implies a high H_2_ evolution rate, with consequent detrimental gas pocket formation around the implant and alkalization occurring in the vicinity of the corroding surface as possibly being deleterious for the surrounding biological environment.^9^

The simplest way to slow down corrosion of Mg and Mg alloys is to form a coating on the magnesium substrate to provide a barrier toward the contact between the substrate and the environments. There are many coating technologies which can be used to coat the magnesium substrate,^10^ such as chemical conversion coating,^11−13^ physical vapor deposition,^14,15^ laser surface treatment,^15,16^ and anodic oxidation.^17−20^ Among these technologies, the latter is one of the most effective and popular methods, even if the growth of protective anodic layers on Mg and Mg alloys is difficult due to the unfavorable Pilling–Bedworth ratio (PBR) for MgO. The latter is defined as the ratio of the volume of the elementary cell of a metal oxide to the volume of the elementary cell of the corresponding metal oxidized to produce the oxide, that is:^21^
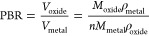
1where *V* is the molar volume, *M* is the molecular weight, ρ is the density, and *n* is the number of metal moles for 1 mole of formed oxide. The oxide layer would be unprotective if the PBR is less than unity because the film that forms on the metal surface is porous and/or cracked, as in the case of magnesium. Thus, in order to enhance the corrosion resistance of Mg and Mg alloys by anodic oxide layers, incorporation from the electrolytic bath of species other than O^2–^ during the anodizing process must be promoted. The anodizing occurs according to a high-field mechanism, thanks to the superimposition of very high electric field (in the order of a few MV cm^–1^), which allows a solid-state migration process of the oxidized metallic cations from the substrates as well as of the anion from the electrolyte, namely, O^2–^ in aqueous solution derived by water deprotonation. Other anions added to the electrolytic solution can also be incorporated even if in a smaller extent than oxygen ions highly available in an aqueous solution. Replacing water as a solvent as well as adding anions that can favor the biocompatibility of the coating is a promising strategy.

**Figure 1:**
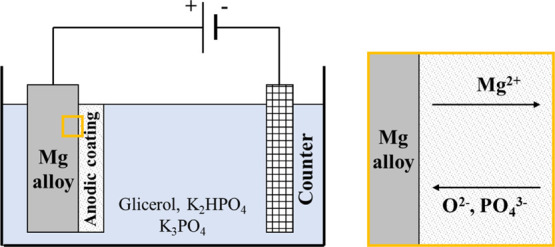
Sketch of the anodizing process setup. Inset: Movement of metallic cations and oxygen anions during anodic coating growth.

In the present work, anodizing of the AZ31 alloy was carried out in the hot glycerol (HG) electrolyte containing K_2_HPO_4_ and K_3_PO_4_, in an attempt to induce the growth of a magnesium phosphate protective layer on the surface of AZ31 with a more favourable PBR than that of MgO. The design strategy for the preparation of these coatings is schematically described in Figure 1.

In the attempt to seal the pores of the anodic layers, to improve their biocompatibility, and to tune the biodegradation rate, the growth of hydroxyapatite (HAP) on the sample surface was induced by a dip coating procedure. The structure and composition of the resultant films were studied by scanning electron microscopy (SEM), energy-dispersive X-ray spectroscopy (EDS), and Raman spectroscopy before and after the immersion. The corrosion resistance of the resulting composite coatings was characterized in Hank’s solution (HS) at 37 °C by open circuit potential (OCP) measurements, electrochemical impedance spectroscopy (EIS), and by recording polarization curves. Accurate measurement of the hydrogen evolution (HE) was obtained by a method based on the measurement of the hydrostatic force resulting from the accumulation of hydrogen in a submerged container.^22^*In vitro* studies were carried out to evaluate the cytocompatibility of the anodized AZ31 samples.

## 2 Materials and Methods

### 2.1 Materials

Composition of the magnesium AZ31 alloy is reported in Table 1.

**Table 1:** Composition (wt %) of the As-Received Mg AZ31 Alloy

Al	Zn	Mn	Si	Fe	Cu	Ni	Mg
2.89	0.92	0.05	0.01	0.004	0.002	0.001	balance

### 2.2 Preparation of AZ31 Coatings and the Anodizing Process

Before any measurements, AZ31 samples were mechanically polished with SiC abrasive papers with grit sizes between P1000 and P2400. Afterward, all samples were cleaned with acetone in an ultrasonic cleaner for 5 min and then rinsed with distilled water.

Anodizing of AZ31 electrodes was carried out in a stirred HG solution containing 0.6 M K_2_HPO_4_ and 0.2 M K_3_PO_4_, with controlled temperature conditions in the galvanostatic unipolar mode (i.e., at a constant current density) using a two-electrode configuration, where the working electrode was the AZ31 sample and the counter electrode was a Pt net with a high surface area. The effect of different operating parameters on the final properties of the coatings was studied: (i) anodizing time (60 s, 30, and 60 min), (ii) bath temperature (100, 160, and 200 °C), and (iii) current density (0.5, 2, and 8 mA cm^–2^). Post anodizing thermal treatment was carried out at 350 °C for 24 h. Each anodic growth was repeated at least three times.

### 2.3 Electrochemical Characterization

To investigate the influence of operating parameters on the properties of the coating on AZ31 samples, OCP, EIS, and polarization curve measurements were carried out using a three-electrode configuration, with an Ag/AgCl/sat. KCl (0.197 V *vs* SHE) reference electrode, while the working and counter electrodes were the same as that for the anodizing process. The characterization electrolyte was the HS, whose composition is reported in Table 2.

**Table 2:** Composition of Hank’s Solution (pH = 6.67)^23^

component	concentration [g L^–1^]
NaCl	8
KCl	0.4
NaHCO_3_	0.35
NaH_2_PO_4_·H_2_O	0.25
Na_2_HPO_4_·H_2_O	0.06
CaCl_2_·2H_2_O	0.19
MgCl_2_	0.19
MgSO_4_·7H_2_O	0.06
glucose	1

The bath temperature was kept constant at 37 ± 1 °C. Impedance spectra were recorded by superimposing to the constant electrode potential (OCP) an a.c. signal of 10 mV in a frequency range between 100 kHz and 100 mHz. Polarization curves were recorded in the electrode potential range between −1.7 V Ag/AgCl and −1.1 V Ag/AgCl with a scan rate of 1 mV s^–1^. All electrochemical measurements were carried out using a PARSTAT 2273 instrument connected to a PC for data acquisition. EIS spectra were then fitted with ZSimpWin software. Each experiment was repeated three times.

### 2.4 SEM, EDX, XRD, and Raman Spectroscopy Analyses

The morphology of the samples was observed using SEM using a Versa 3D microscope (FEI, Thermo Fischer). The cross sections were prepared by mounting the samples vertically in epoxy resin and mechanically grinding near parallel to the coating/substrate interface direction with emery paper down to 2400 grit. Images were taken at a voltage of 30 kV. EDX microanalysis was carried out with a XFlash 6l10 EDX probe (Bruker) integrated in the microscope. The spectra, obtained at the voltage of 30 kV, were processed with Esprit software for qualitative and quantitative analyses. The same software was used for EDX mapping. The maps were obtained at the voltage of 30 kV with an acquisition time of 300 s.

X-ray diffraction (XRD) measurements were performed using a PanAnalytical Empyrean diffractometer with a Cu anode (Cu Kα radiation, λ = 0.15405 nm) equipped with a PIXCel1D detector (voltage: 40 kV, current: 40 mA). The XRD patterns were collected over the 2θ angle range of 10–90°.

Micro-Raman analysis was performed through a Renishaw inVia Raman microscope spectrometer equipped with a microprobe (50×) and a CCD detector with a Nd:YAG laser with a wavelength of 532 nm.

### 2.5 Measurement of H_2_ Evolution during Corrosion in HS

The sample was mounted below a bottle completely immersed in HS. The bottle was closed at the bottom through a deformable diaphragm to allow collection of the evolved hydrogen bubbles. The bottle and the sample were mounted on a support that was hooked into a precision laboratory balance (Mettler Toledo XS1003S). An inert weight was added to the bottle ensuring stable submersion. With this arrangement, when hydrogen evolves from the electrode surface, it is collected by the bottle. Due to the hydrostatic force, the weights of the bottle and sample decrease proportionally to the volume of evolved hydrogen. Each measurement of H_2_ evolution was repeated three times.

### 2.6 Cytotoxicity Tests

For indirect cytotoxicity tests, the samples (coated and not-coated) were rinsed with Milli-Q water and then sterilized by immersion in ethanol for 24 h followed by UV ray treatment for 2 h (1 h per side). Samples were then incubated with Dulbecco’s modified Eagle medium (DMEM, Sigma-Aldrich) at 37 °C for 24 h with a volume/surface ratio of 5 ml/cm^2^.^24^ Afterward, the as-treated media were collected in 50 mL of falcon and utilized for the cytotoxicity test. MC3T3-E1 preosteoblastic cells purchased from Sigma-Aldrich (ECACC) were cultured in DMEM added to 10% fetal bovine serum, 1% glutamine, and 1% antibiotic at 37 °C and in a 5% CO_2_ atmosphere. 10^4^ osteoblastic cells were seeded into the wells of a 24-well culture plate (10 mm diameter) and incubated with normal DMEM at 37 °C and 5% CO_2_. After 24 h, the medium was replaced with treated media. Normal DMEM was used as the positive control to evaluate the effect of coatings.

The proliferation rate was evaluated using an AlamarBlue cell viability reagent (invitrogen). Each well was incubated at 37 °C and 5% CO_2_ for 3 h with 500 μL of AlamarBlue reagent (10×) diluted (1:10) in DMEM. The resulting fluorescence was read on a plate reader at an excitation wavelength of 530/25 (peak excitation is 570 nm) and an emission wavelength of 590/35 (peak of emission is 585 nm). Cytotoxicity assays were carried out after 0, 1, 4, and 7 days of culture. Each cytotoxicity test was repeated three times.

## 3 Results and Discussion

### 3.1 Growth Kinetics and Anodizing Process Optimization

**Figure 2:**
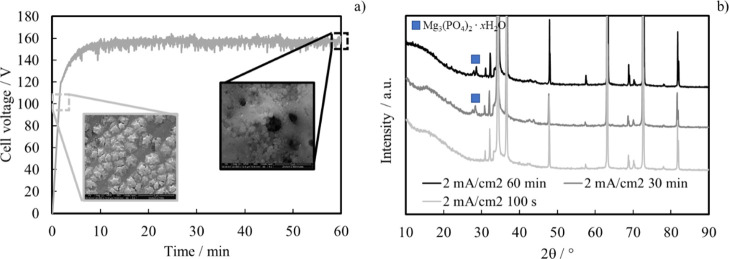
(a) Dependence of cell voltage on time during AZ31 anodizing at 2 mA cm^–2^ for 60 min at 160 °C. Inset: SEM micrographs related to samples anodized for 100 s and 60 min. (b) XRD patterns related to samples anodized at 2 mA cm^–2^ for 100 s, 30 min, and 60 min.

Figure 2 shows the dependence of cell voltage on time recorded during the anodizing of AZ31 carried out galvanostatically at 2 mA cm^–2^ at 160 °C for 60 min in HG solution.

The effect of anodizing time on the properties of the coating was studied by interrupting the anodizing process at 100 s, 30 min, and 60 min. The cell voltage rises linearly during the early stage of anodic polarization with a slope of ∼1.2 V s^–1^ up to ∼120 V, followed by a much slower increase accompanied by oscillations due to the onset of dielectric breakdown phenomena. The slope, d*V*/d*t*, of the linear part of the curve can be linked to the film growth parameters according to the following relationship^18^
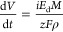
2where *i* is the anodic current density, *E*_d_ is the electric field strength, *M* is the molecular weight of the film constituent, *z* is the number of electrons, *F* is the Faraday constant, and ρ is the film density. As described in detail in Appendix A, eq 2 allows us to estimate the barrier layer thickness corresponding to two limiting cases, that is, 110 nm assuming the formation of MgO (no foreign anions are incorporated from the electrolyte apart O^2–^ anions) and 335 nm assuming the formation of Mg_3_(PO_4_)_2_ [only (PO_4_)^3–^ anions are incorporated from the electrolyte]. The second part of the anodizing process allowed the formation of a thicker coating, thanks to the formation in a hard anodizing regime of an outer porous layer.^25^

**Figure 3:**
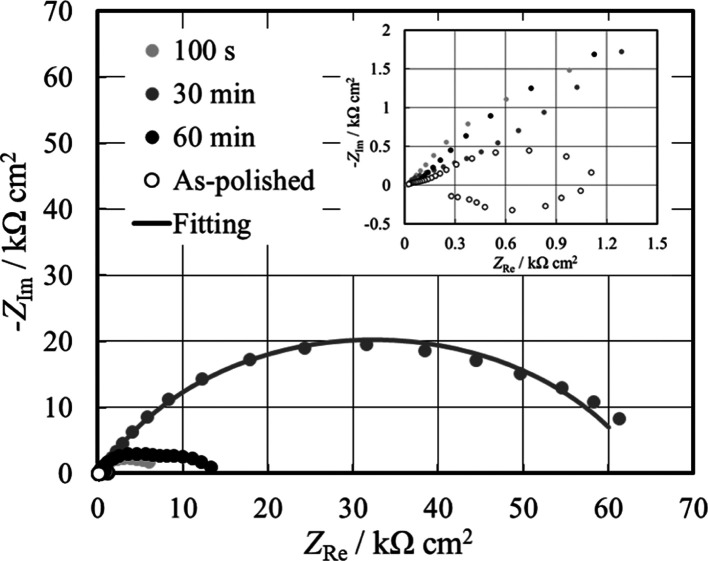
EIS spectra in the Nyquist representation recorded at OCP in HS for the as-polished and anodized AZ31 samples at different times. Inset: Zoom of EIS spectra at low real and imaginary parts. Continuous lines: fitting lines.

Figure 3 shows the EIS spectra recorded for anodized AZ31 samples in HS at 37 °C at the OCP after 1 h of immersion. For comparison, the EIS spectrum recorded for as-polished AZ31 is also reported. The spectra in the Nyquist representation for the coated alloy look like depressed semicircles.

**Figure 4:**
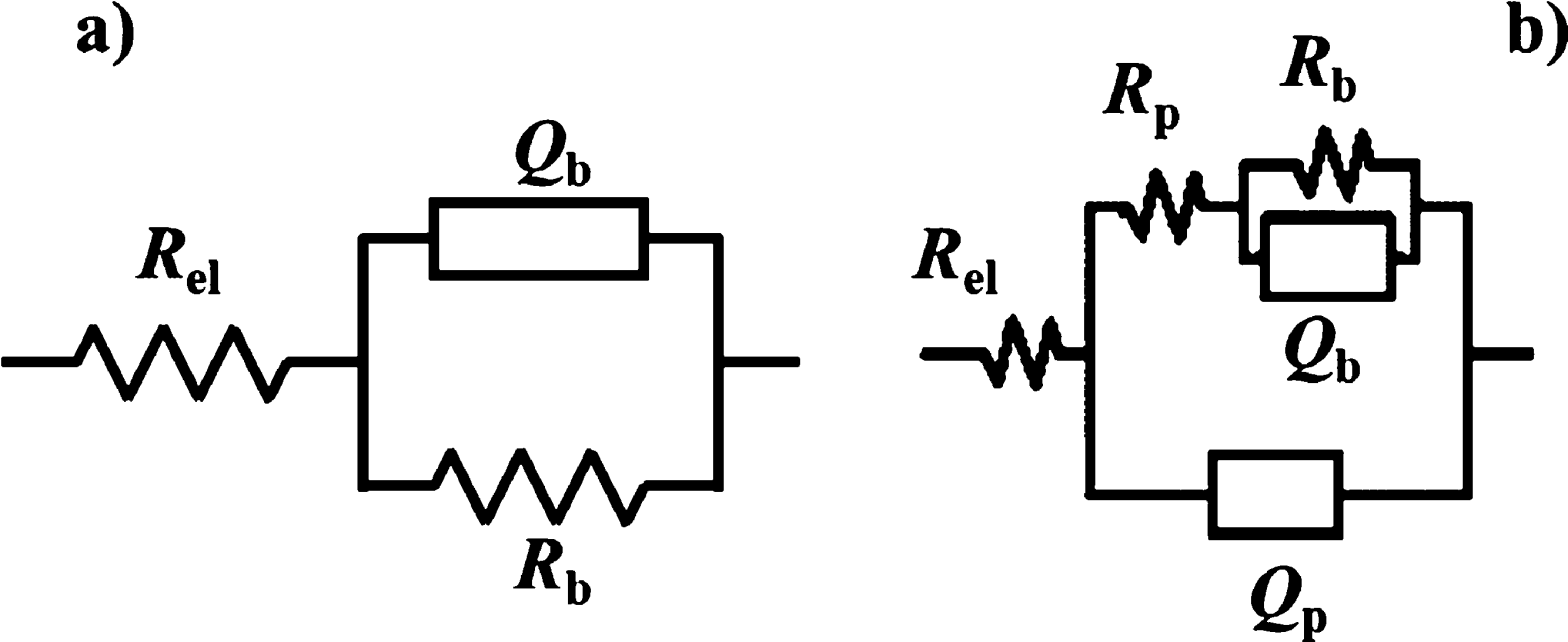
EECs used to model the electrochemical behavior of the systems comprising (a) only the barrier layer and (b) barrier + porous layers.

It is interesting to note that two different electrical equivalent circuits (EECs) were used to fit EIS spectra, and they are shown in Figure 4.

In fact, in the case of the AZ31 sample anodized for 100 s, best fitting was reached using an EEC comprising just one time constant, that is, a parallel (*R*_b_*Q*_b_) in series with *R*_el_ (electrolyte resistance), where *Q* is a constant phase element modelling the nonideal capacitance of the single-layer coating. This is due to the barrier nature of the anodic film grown for 100 s, and the use of the EEC, as shown in Figure 4a, is in agreement with that reported in the literature, for which the electrochemical behavior of barrier-like anodic films can be successfully modelled by a simple parallel *RQ*.^26−28^ Conversely, in the case of AZ31 samples anodized for longer times (e.g., 30 and 60 min), a more complex EEC is needed to model the electrochemical behavior of anodic films immersed in HS. In particular, the EEC used is shown in Figure 4b, where the parallel (*R*_b_*Q*_b_) is in series with *R*_p_ representing the outer layer resistance, that is, the electrolyte resistance inside the pores of the anodic films. This part of the electrical circuit is in parallel with a *Q*_p_ element representing the outer porous layer (nonideal) capacitance. It was then added in series as a final element with the electrolyte resistance *R*_el_. Best fitting parameters, estimated by using EECs, as shown in Figure 4a,b, are reported in Table 3.

**Table 3:** Fitting Parameters Related to EIS Spectra Shown in Figure 3

anodizing time	*R*_el_ [Ω cm^2^]	*R*_p_ [kΩ cm^2^]	*Q*_p_ [S s^n^ cm^–2^]	*n*	*R*_b_ [kΩ cm^2^]	*Q*_b_ [S s^*n*^ cm^–2^]	*n*	χ^2^
100 s	15				7	2.0 × 10^–5^	0.76	2.5 × 10^–3^
30 min	15	0.6	4.9 × 10^–7^	0.76	64	2.5 × 10^–6^	0.71	7.4 × 10^–4^
60 min	15	10	8.2 × 10^–6^	0.70	3	1.5 × 10^–4^	1	1.1 × 10^–2^

The highest overall resistance corresponds to an anodizing time of 30 min, suggesting that the corrosion rate is slowed down synergistically by both the barrier inner layer and the porous outer layer. The formation of anodic films during spark anodizing is more complex than the growth of the anodic film under high field mechanism. As reported above (see eq 2), in the latter case, the thickness of the barrier film is directly proportional to the applied voltage. This statement is no longer true for anodizing occurring under sparking regime since thickening of the inner (so called dense layer) and outer porous layers is determined by several types of discharge and by plasma reactions occurring as a consequence of the discharges themselves.^29^ The fitting of EIS spectra suggests that a longer duration of the sparking anodizing may induce damage in the inner layer and induce the formation of a thicker porous layer with a detrimental effect on the corrosion resistance.

It is interesting to note, by looking at the inset of Figure 3, that an inductive loop is present in the low-frequency range of the EIS spectrum of as-polished AZ31. The presence of an inductive loop in the EIS spectrum is typically related to the electrochemical processes that involve more than one reaction step, where one step comprises the formation of adsorbed species on the surface leading to a dependence of reaction kinetics on the electrode surface coverage.^26,30,31^ This behavior is usually observed in the case of pure Mg and Mg-alloy electrodes, for which the oxide/hydroxide film that spontaneously grows on the surface is quite incoherent and does not cover all of the substrate surface, not offering good corrosion protection to the metal, mostly in acidic or neutral environment, as HS. Moreover, the cathodic process usually coupled to the Mg oxidation is the water reduction that leads to gaseous HE that typically occurs following a multistep kinetic path, involving the presence of adsorbed species.^32,33^

**Figure 5:**
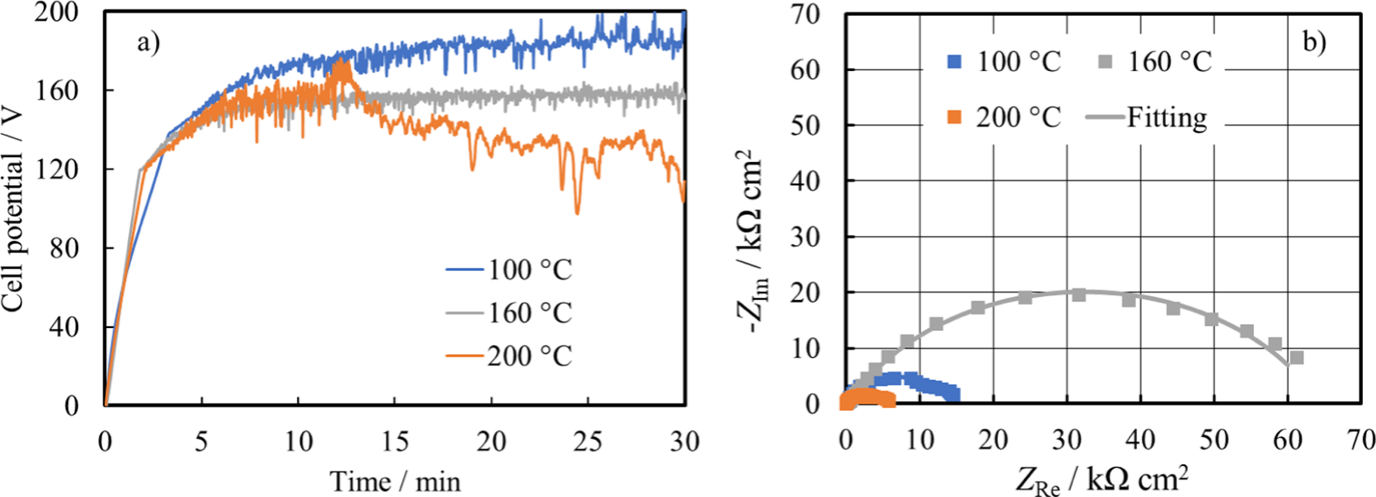
(a) Dependence of cell potential on time during AZ31 anodizing at 2 mA cm^–2^ for 30 min at different temperatures. (b) EIS spectra in the Nyquist representation recorded at OCP in HS for anodized AZ31 samples at different temperatures. Continuous lines: fitting lines.

Anodizing process was also carried out at different temperatures (i.e., 100 and 200 °C), as shown in Figure 5a, where the comparison of the growth curves is reported for a galvanostatic polarization at 2 mA cm^–2^ for 30 min.

Regardless of the anodizing bath temperature, the cell voltage followed the same dependence on time shown in Figure 2, that is, an initial linear dependence related to the growth of a barrier layer followed by voltage oscillations due to sparking phenomena. It is noteworthy to mention that the slope of the linear part of the growth curve strongly depends on the bath temperature, suggesting a different growth efficiency and leading to a different structure and/or a different barrier layer thickness (see eq 2). Moreover, at the highest temperature (namely 200 °C), the dissolution rate became too high to keep the final formation voltage (as in the case of growth at 100 and 160 °C), with a decrease of cell voltage value for *t* > 12 min. In spite of the corrosion potential values being the same regardless of the anodizing bath temperature (−1.48 V *vs* Ag/AgCl for the anodic film grown at 160 °C and −1.50 V *vs* Ag/AgCl for the coatings grown at 100 and 200 °C), a clear difference in the electrochemical behavior could be assessed by looking at the EIS spectra, recorded at OCP, as shown in Figure 5b. For the fitting procedure, EEC was used, as shown in Figure 4b, since the structure of the coating obtained for 30 min was porous, regardless of the bath temperature during the anodic film growth. EIS fitting parameters are reported in Table 4.

**Table 4:** Fitting Parameters Related to EIS Spectra Shown in Figure 5b

anodizing temperature	*R*_el_ [Ω cm^2^]	*R*_p_ [kΩ cm^2^]	*Q*_p_ [S s^n^ cm^–2^]	*n*	*R*_b_ [kΩ cm^2^]	*Q*_b_ [S s^*n*^ cm^–2^]	*n*	χ^2^
100 °C	17	0.20	5.3 × 10^–6^	0.69	14	1.6 × 10^–6^	0.89	4.8 × 10^–3^
160 °C	15	0.6	4.9 × 10^–7^	0.76	64	2.5 × 10^–6^	0.71	7.4 × 10^–4^
200 °C	13	0.06	2.2 × 10^–5^	0.63	5.7	3.3 × 10^–6^	0.93	2.4 × 10^–3^

The best performance, in terms of overall system resistance, was shown by the coating grown at 160 °C, while the worst one was associated with the coating grown at 200 °C (*R*_b_ = 5.7 kΩ cm^2^), as the temperature of the anodizing bath plays a major role in the quality of the coating grown on AZ31 samples.

**Figure 6:**
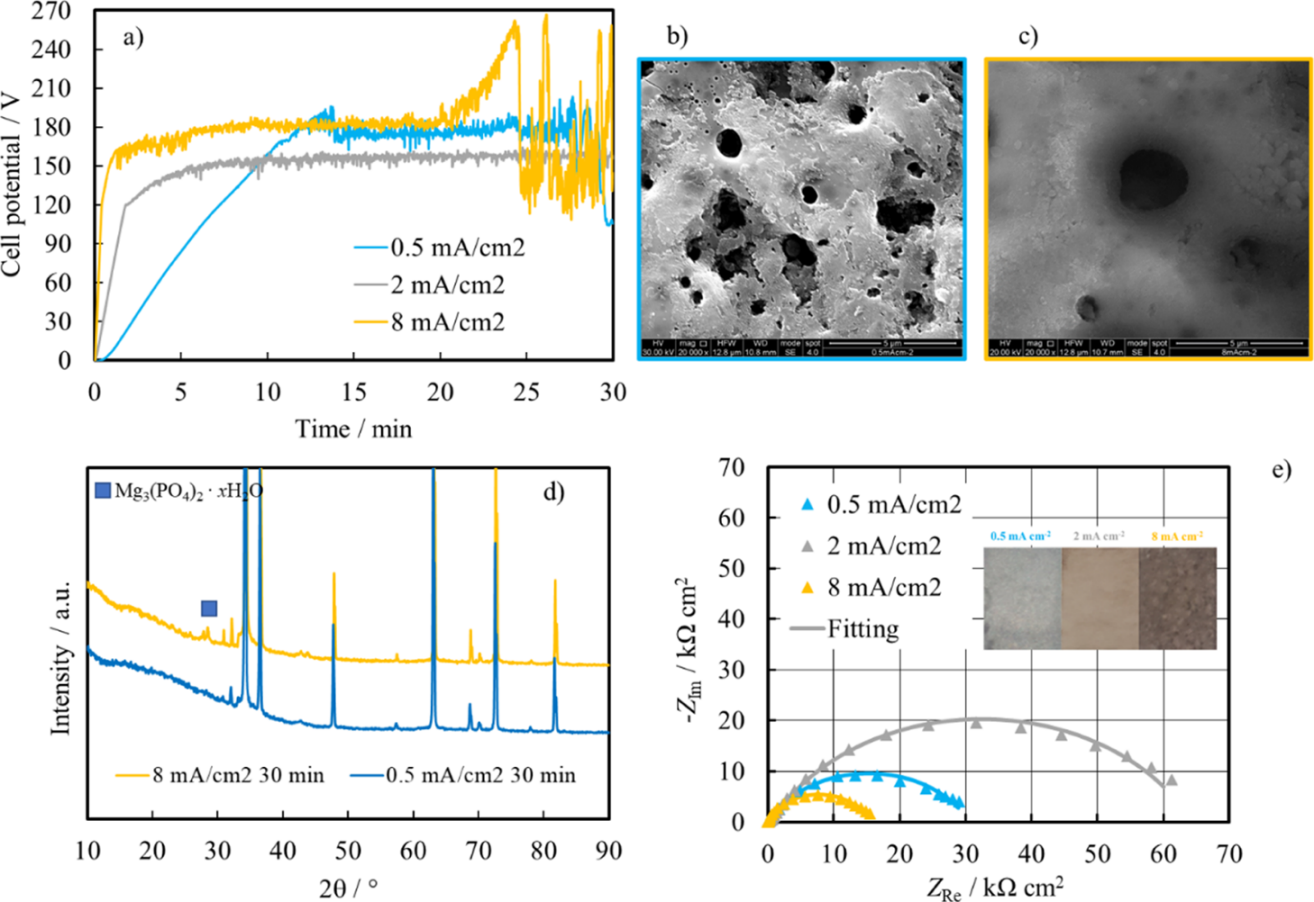
(a) Dependence of the cell potential on time during AZ31 anodizing at 160 °C for 30 min at different current densities. SEM micrographs related to samples anodized at 160 °C for 30 min at (b) 0.5 and (c) at 8 mA cm^–2^ with corresponding (d) XRD patterns. (e) EIS spectra in the Nyquist representation recorded at OCP in HS for anodized AZ31 samples at different current densities. Continuous lines: fitting lines. Inset: Surface of the AZ31 coupons soon after anodizing at different current densities.

AZ31 coupons were also anodized for 30 min at 0.5, 2, and 8 mA cm^–2^ at 160 °C in order to assess the effect of current density on the quality of the prepared coatings (see Figure 6a). As expected, the higher the slope of the voltage–time curve, the higher the applied current density, and at 8 mA cm^–2^, the voltage increases significantly for a long anodizing time probably due to intense discharges, as confirmed by the SEM images (see Figure 6c) showing the formation of wider pores when AZ31 is anodized at this high current density. Notably, the XRD patterns for the coating grown at 8 mA cm^–2^ confirmed the presence of crystalline Mg_3_(PO_4_)_2_ (according to ICSD card: 020796, see Figure 6d), whose reflections are missing for the coating grown at 0.5 mA cm^–2^ probably due to the formation of a thinner and/or less crystalline layer. Figure 6e shows the EIS spectra recorded in HS at 37 °C after 1 h of immersion at the OCP. As for all samples anodized for 30 min, the best EIS spectra fitting was obtained by using the EEC that comprise the presence of an outer porous layer in the structure of the coatings.

The highest resistance corresponds to an anodizing current density of 2 mA cm^–2^, while the worst result was shown by the AZ31 sample anodized at 8 mA cm^–2^ (see best fitting parameters reported in Table 5).

**Table 5:** Fitting Parameters Related to EIS Spectra Shown in Figure 6e

anodizing current density	*R*_el_ [Ω cm^2^]	*R*_p_ [kΩ cm^2^]	*Q*_p_ [S s^*n*^ cm^–2^]	*n*	*R*_b_ [kΩ cm^2^]	*Q*_b_ [S s^*n*^ cm^–2^]	*n*	χ^2^
0.5 mA cm^–2^	20	0.16	3.1 × 10^–7^	0.81	31	4.8 × 10^–6^	0.70	9.6 × 10^–4^
2 mA cm^–2^	15	0.6	4.9 × 10^–7^	0.76	64	2.5 × 10^–6^	0.71	7.4 × 10^–4^
8 mA cm^–2^	20	15	7.2 × 10^–6^	0.79	1.4	7.7 × 10^–4^	1	6.5 × 10^–3^

The surface of the AZ31 coupons soon after anodizing is uniformly covered by a coating, whose color is a function of the anodizing current density (see inset of Figure 6e). From the analysis reported in this section, best results, in terms of corrosion resistance in HS, were shown by AZ31 samples anodized at 2 mA cm^–2^ for 30 min with an anodizing bath temperature of 160 °C.

**Figure 7:**
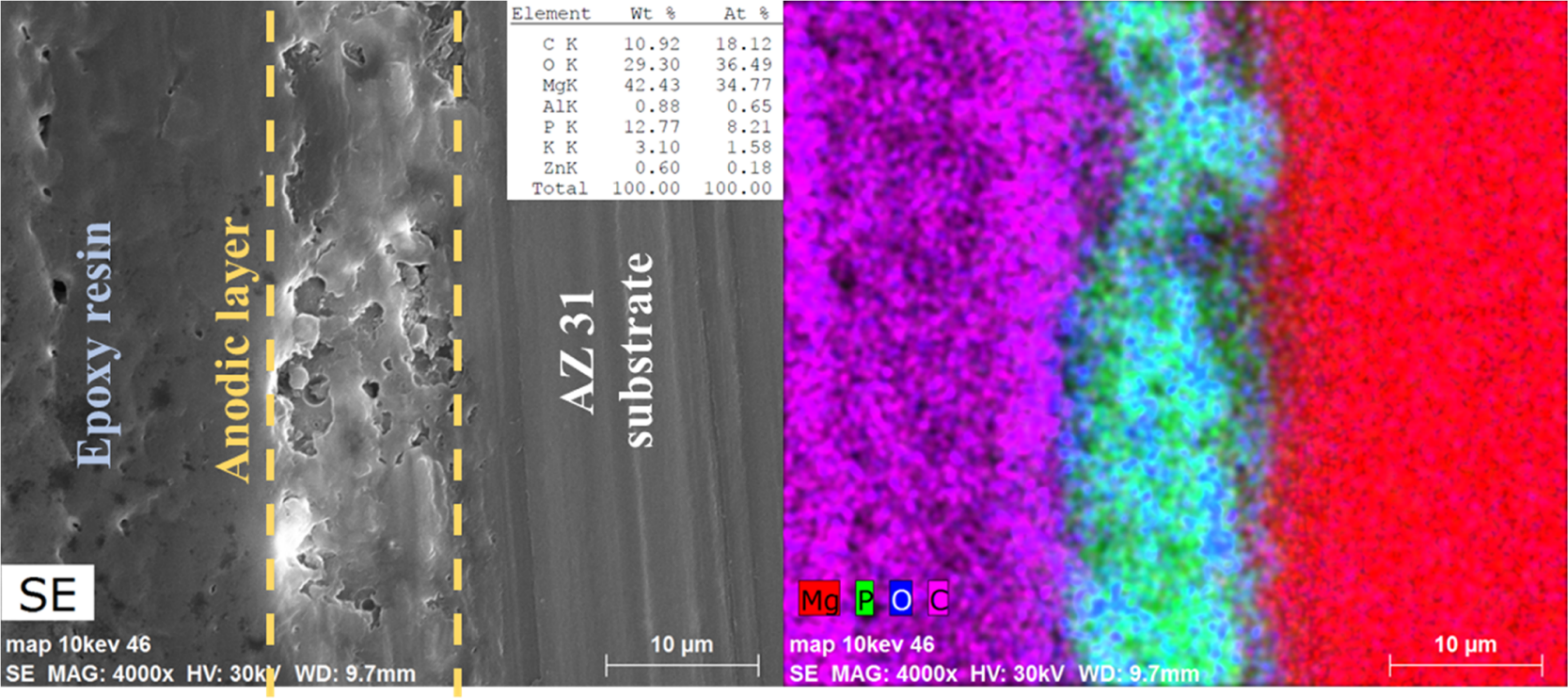
SEM cross section of the AZ31 sample anodized in the optimized anodizing conditions with the associated EDS map of the elements contained in the anodic coating.

Figure 7 shows the SEM cross section of an AZ31 sample anodized in the optimized anodizing conditions with the associated EDS map of the elements contained in the anodic film.

The coating thickness resulted to be ∼12 μm. From the elemental analysis (at. %), it is possible to assess the composition of the coating on the AZ31 sample that is very close to that of Mg_3_(PO_4_)_2_, with P and O quite uniformly distributed across the anodic layer.

### 3.2 Post Anodizing Annealing Process

**Figure 8:**
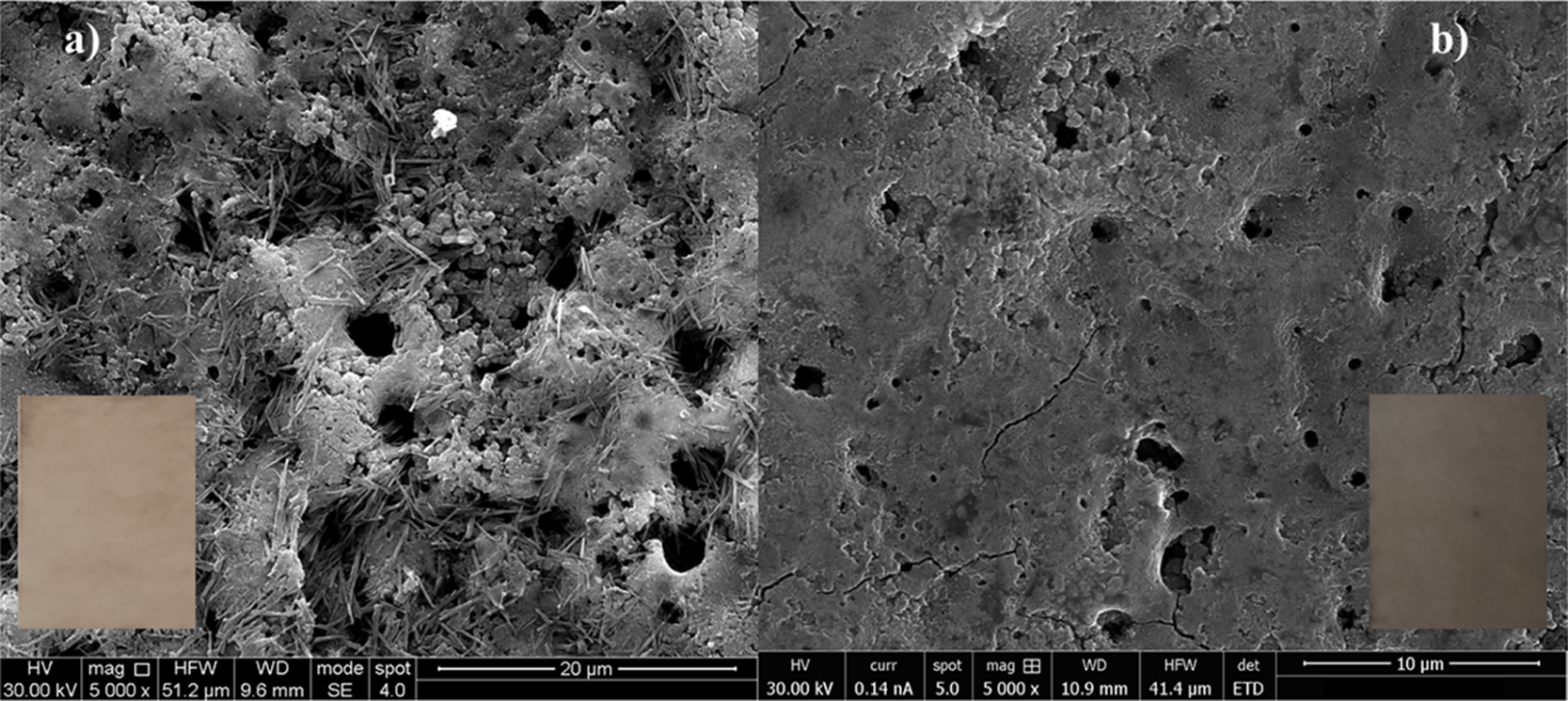
Scanning electron micrographs showing the morphologies of anodic coatings formed on AZ31 samples (a) before and (b) after thermal treatment. Inset: Surface of the anodic coatings.

Once the anodizing process was optimized in terms of current density, anodizing time, and bath temperature, we studied the effect of a post anodizing annealing process on the coatings’ morphology and on their corrosion resistance. AZ31 coupons anodized at 2 mA cm^–2^ for 30 min at 160 °C were annealed under air exposure at 350 °C for 24 h. The effect of the thermal treatment on the coating microstructure was evaluated by SEM. Figure 8a,b shows the surface morphology of the as-anodized and annealed coupons, respectively, while the insets show the color of the coatings before and after the thermal treatment. Moreover, the thermal treatment leaves the sample surface free of the deposits present onto the sample soon after the anodizing process.

**Figure 9:**
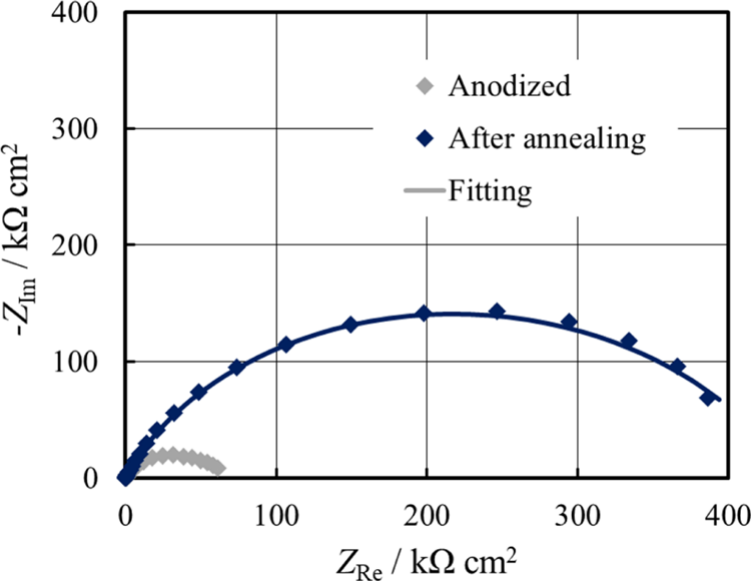
EIS spectra in the Nyquist representation recorded at OCP in HS for anodized AZ31 samples before and after the thermal treatment. Continuous lines: fitting lines.

Thermal treatment also had an effect on the corrosion resistance of the alloy samples, as better evaluated by EIS. EIS spectrum of the annealed coupon recorded in HS at 37 °C after 1 h of immersion is shown in Figure 9 and best fitting parameters (see Table 6) were obtained by using the EEC, as shown in Figure 4b.

**Table 6:** Fitting Parameters Related to EIS Spectra Shown in Figure 9

sample	*R*_el_ [Ω cm^2^]	*R*_p_ [kΩ cm^2^]	*Q*_p_ [S s^*n*^ cm^–2^]	*n*	*R*_b_ [kΩ cm^2^]	*Q*_b_ [S s^*n*^ cm^–2^]	*n*	χ^2^
as-anodized	15	0.6	4.9 × 10^–7^	0.76	64	2.5 × 10^–6^	0.71	7.4 × 10^–4^
annealed	3.6	2.1	2.3 × 10^–7^	0.83	440	4.3 × 10^–7^	0.64	5.2 × 10^–4^

The corrosion resistance was enhanced by one order of magnitude due to thermal treatment with respect to that estimated for AZ31 soon after anodizing. A more compact morphology is representative of a kind of sealed structure that diminishes the contact of the underneath barrier layer, with highly aggressive HS leading to a reduction of corrosion phenomena.

We also evaluated the effect of longer immersion time in HS on the composition of the coating, especially in terms of at. % of Ca and P, that is, the main elements of HAP compound. At. % obtained by EDX analysis is reported in Table 7 as a function of the immersion time from 3 to 14 days.

**Table 7:** At. % of Ca and P Components in the Anodized AZ31 Sample after Different Immersion Times in HS

immersion time [days]	Ca [at. %]	P [at. %]	Ca/P ratio
3	6.04	15.33	0.39
7	9.04	14.92	0.61
14	13.02	16.98	0.77

**Figure 10:**
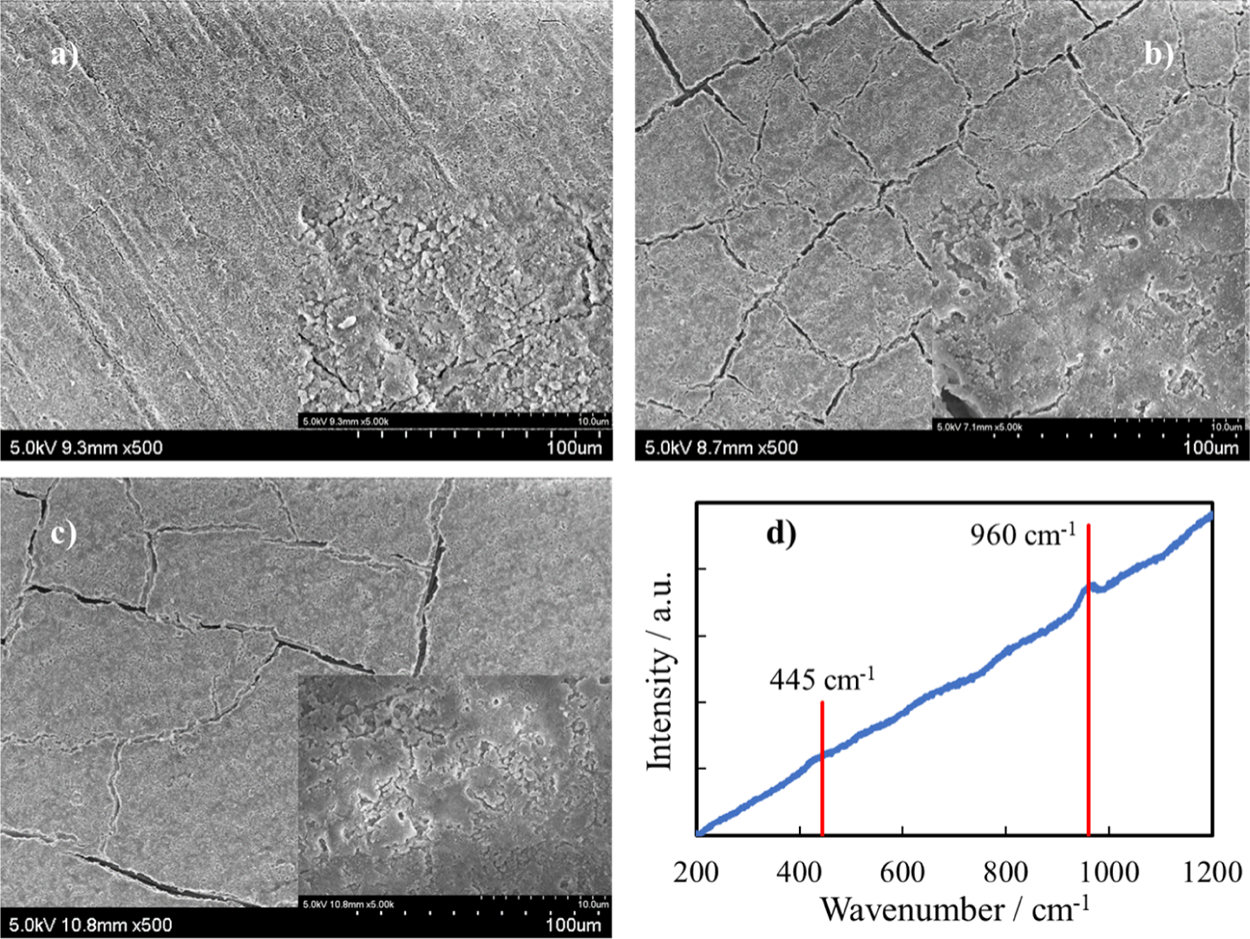
Scanning electron micrographs showing the morphologies of anodic coatings, after annealing, formed on AZ31 samples after (a) 3 days, (b) 7 days, and (c) 14 days of immersion in HS. (d) Raman spectrum related to the anodic coating after 14 days of immersion in HS.

The ratio Ca/P increases by increasing the immersion time in HS, from 0.39 to 0.77. These values are quite far from the ratio Ca/P contained in HAP, that is, 1.67, but are affected by the high P signal due to the P incorporated into coatings during the anodizing process. The effect of immersion in HS was also assessed by looking at the morphology of anodized AZ31 samples that is shown in Figure 10 for the studied immersion times.

After 3 days of immersion (see Figure 10a), the surface looked still compact as that shown in Figure 8b, while a classical “mud” structure developed by increasing the immersion time in HS (i.e., 7 and 14 days, see Figure 10b,c, respectively). Cracks present on the surface of samples can be explained by taking into account (i) the residual-stress release during the hard anodizing process when the coating is immersed in HS, (ii) the HE due to the corrosion of magnesium alloy,^34,35^ and/or (iii) the penetration of HS into anodic layer pores and the following formation of HAP with higher volume.^35^ The formation of HAP is confirmed also by the Raman spectrum, as shown in Figure 10d, related to the anodized AZ31 sample immersed for 14 days in HS. In particular, PO_4_^3–^ features in HAP are characterized by the ν_1_ symmetric stretching (P–O) mode at 960 cm^–1^ and by the ν_2_ bending (O–P–O) mode at about 430–450 cm^–1^.^36,37^

### 3.3 Evaluation of H_2_ Evolution Rate

A significant issue about the usage of magnesium alloys in biomedical applications is that the HE reaction (HER) is coupled to the Mg corrosion (dissolution) process according to the following reaction
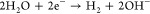
3

that is, water reduction reaction (WRR). Actually, oxygen reduction reaction can also be considered coupled to the Mg corrosion process, but at the corrosion potential of Mg alloys, WRR has to be considered as the dominant cathodic reaction. Gaseous HE could be dangerous depending on its evolution rate. If it is sufficiently high, hydrogen bubbles can accumulate in gas pockets next to the implant, which can lead to a delay in the healing of the surgery region and/or even to the necrosis of tissues.^38^ In the worst case, hydrogen bubbles can also be a serious issue for the blood circulating system. On the contrary, if H_2_ generation is sufficiently slow, gas can be transported away from the implant site avoiding any possible gas build-up. Another issue related to reaction 3 is the environment alkalization that, although could be beneficial for the passivation of Mg alloy surface, can also lead to cell death.^39^

**Figure 11:**
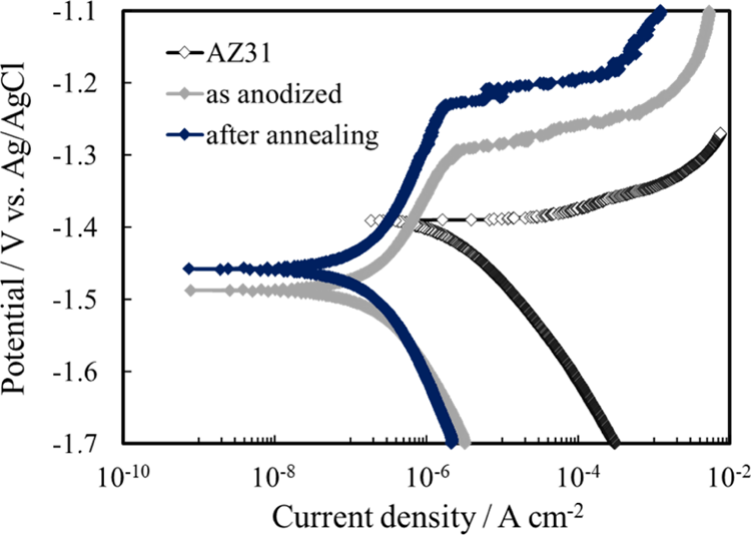
Polarization curves, recorded at 1 mV s^–1^, for as-polished, anodized, and after annealing AZ31 samples.

For these reasons, it is crucial to study HER and to measure the amount of hydrogen evolved. Typically, potentiodynamic measurements are carried out in order to estimate the corrosion current density, *i*_corr_, and therefore, the amount of H_2_ generated in the condition of free corrosion in the HS electrolyte. In Figure 11 polarization curves recorded for as-polished AZ31, anodized, and thermal treated samples are shown.

**Figure 12:**
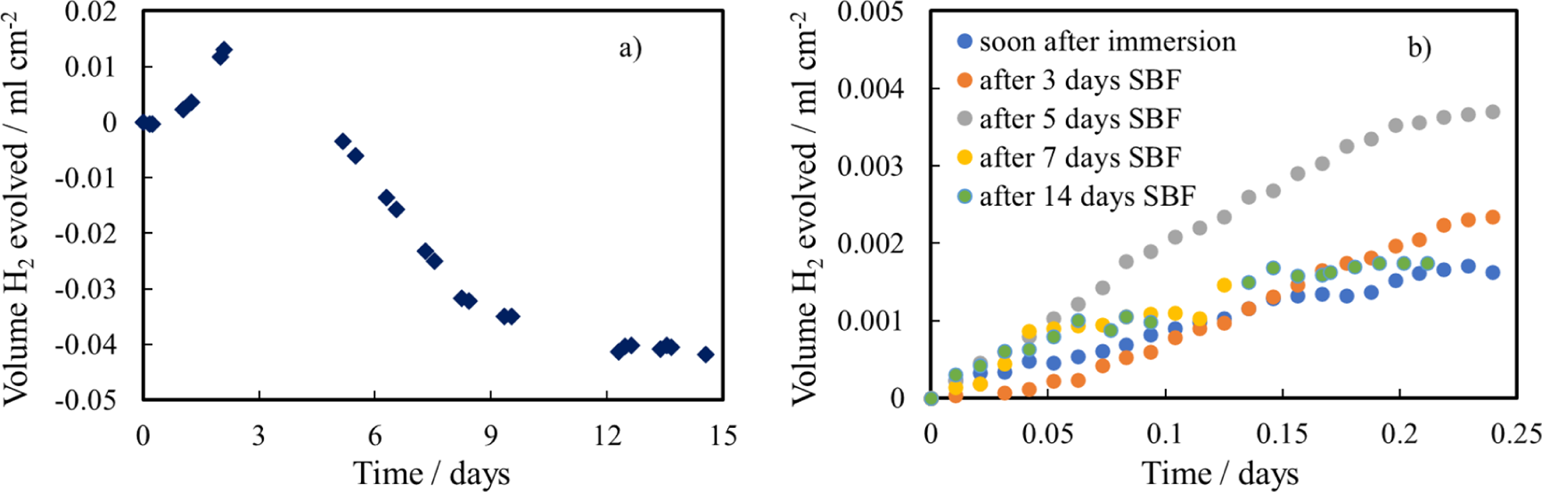
(a) Volume of hydrogen evolved in 15 days for an anodized and thermal treated AZ31 sample. (b) Volume of hydrogen evolved for an anodized and thermal treated AZ31 sample after different immersion times in HS.

The thermal treated sample showed the lowest cathodic current density, the lowest passivity current density, and the highest pitting potential. The highest *i*_corr_ and, thus, the cathodic current density was measured for the as-polished AZ31 sample. Actually, the electrochemical estimate of *i*_corr_ from the polarization curves of Mg and Mg alloys suffers from the uncertainty due to the negative difference effect or the anomalous HE phenomenon.^9,30,40,41^ In fact, when Mg and Mg alloys are polarized anodically with respect to their corrosion potential, higher dissolution rates are recorded, but the HER rate increases by increasing the anodic polarization. Therefore, a more accurate method to estimate the HE rate is mandatory. In this regard, we used the method described by Strebl and Virtanen that is based on the simple measurement of the hydrostatic force resulting from the accumulation of hydrogen in a submerged container (see Section 2.4 for experimental details).^22^ As expected, hydrogen evolved during the measurement time led to a decrease in the recorded weight by the instrument due to a buoyant force that, according to the Archimedes’ principle, is equal to the weight of the displaced fluid volume. The amount (volume) of hydrogen evolved as function of time can be derived according to the following equation^22^
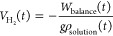
4where *W*_balance_ is the recorded weight, *g* is the gravitational acceleration, and ρ_solution_ is the density of the HS electrolyte. The volume of hydrogen evolved normalized for the sample area as a function of time is then reported in Figure 12a.

As it is possible to see, the volume of hydrogen evolved increases during the first 50 h of measurements and then decreases (i.e., recorded weight by the balance increases). This is probably due to the dissolution of gaseous H_2_ into the solution, thus not exerting a buoyant force anymore. Because of this phenomenon, this technique seems not suitable to give reliable results about the amount of hydrogen evolved for long-run measurements. To overcome this issue, a discontinuous measurement of the volume of evolved H_2_ was carried out: the sample of anodized and thermal treated AZ31 was immersed in HS and, after a certain time, was removed from that solution and immersed in HS for the HE rate evaluation setup. Then, the weight was recorded for about 6 h before the sample was immersed again in another beaker containing HS. In this way, we reached longer measurement times than those of continuous measurement. Figure 12b shows the volume of hydrogen evolved for the discontinuous measurement at different immersion times. The rate of HE was 0.007 mL cm^–2^ day^–1^ soon after the immersion in HS, then 0.011, 0.016, 0.010, and 0.008 mL cm^–2^ day^–1^ after 3, 5, 7, and 14 days, respectively. These HE rates were lower or of the same order of magnitude than 0.01 mL cm^–2^ day^–1^ that was reported as the tolerated rate to consider the treated AZ31 sample as the candidate biodegradable material for further tests in the human body.^38^ The highest recorded HE rate resulted, in any case, lower or of the same order of magnitude of other HE rates reported in the literature for Mg and Mg-based electrodes for biomedical devices.^42−45^

### 3.4 Cytotoxicity Tests

**Figure 13:**
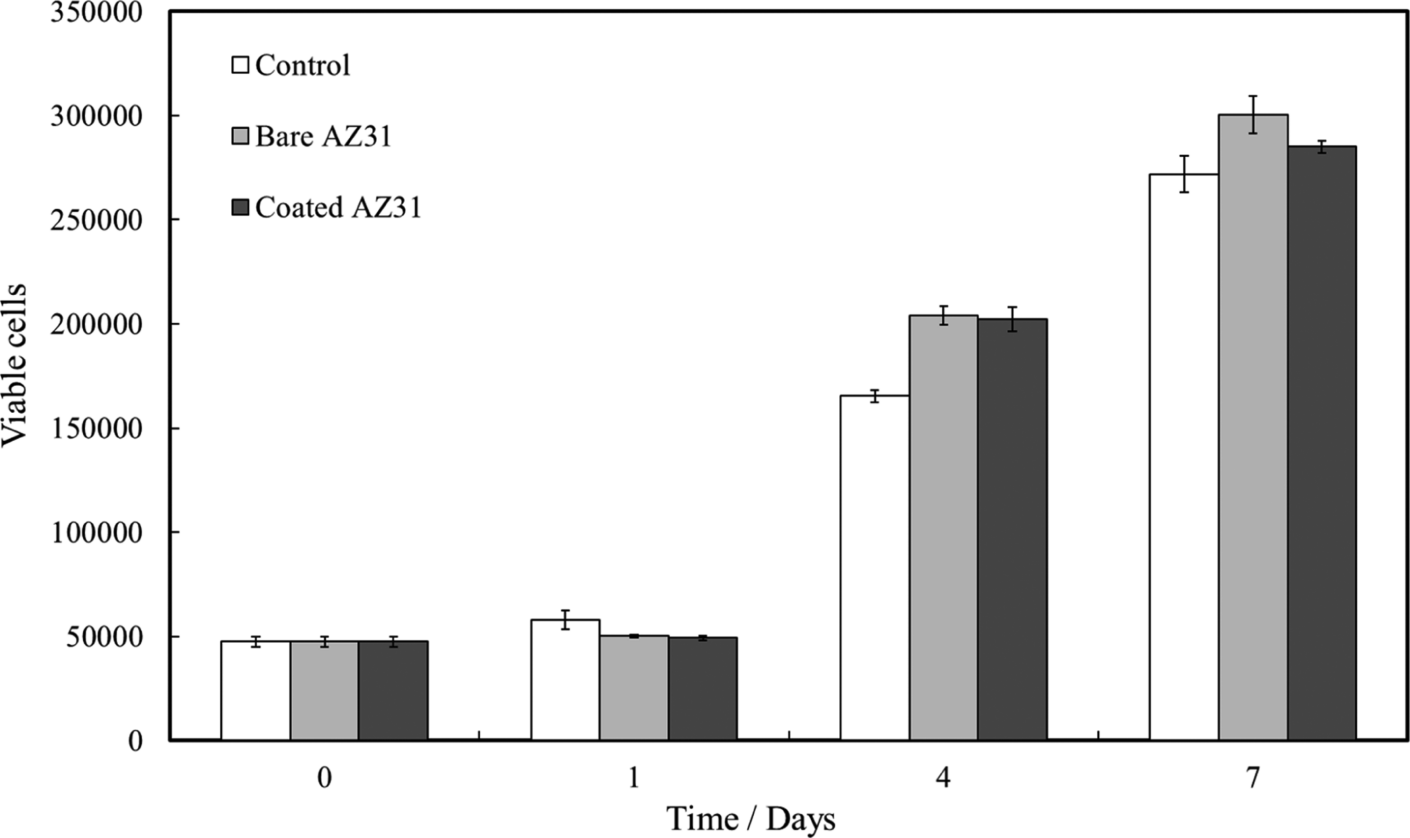
Viability of MC3T3-E1 cells grown with not-treated medium (control) and bare and coated AZ31 samples.

Finally, we investigated the cytotoxicity of the anodized and thermal treated AZ31 samples that are possible candidates for biomedical applications. Cytotoxicity tests were carried out *in vitro* using the preosteoblastic cell line, MC3T3-E1. Cell culture was carried out using media derived from incubation with samples for 24 h at an established volume/surface ratio. Cell viability assays were based on following the growth up to 7 days of culture, whose results are shown in Figure 13.

From the graph, it can be observed that in all three cases, the number of cells increases by increasing the time [from 5 × 10^4^ (seeding days) to about 3 × 10^5^ (7 days)], without statistically significant difference among the samples at each time point. Furthermore, it is important to underline that in all cases, the viability of the cells grown with the incubated media was abundantly over 70% with respect to the control viability, thus confirming the noncytotoxicity of the tested materials, according to the followed ISO standard.^46^

## 4 Conclusions

Anodizing of AZ31 Mg alloys was performed in HG electrolyte containing K_2_HPO_4_ and K_3_PO_4_ salts to grow a corrosion resistant and biocompatible coating that can be used in biomedical devices. Anodic film onto the metallic substrate had a two-layer structure, that is, an inner barrier layer and a thicker outer porous layer. The as-formed coatings were characterized in HS in order to verify their compatibility with the human body environment. The anodizing process was optimized in terms of a suitable current density (2 mA cm^–2^), process time (30 min), and bath temperature (160 °C) to maximize the corrosion resistance of AZ31 coupons. A thermal treatment at 350 °C for 24 h was also performed further enhancing the samples’ corrosion resistance, sealing the outer porous layer, making it more compact, and thus reducing the contact time between the substrate and the aggressive environment, that is, HS.

The amount of hydrogen evolved from corrosion of AZ31 samples was measured with a simple gravimetric method rather than estimated from electrochemical potentiodynamic measurements. H_2_ evolution rate resulted to be lower than the tolerated rate to consider samples as candidate biodegradable materials. Cytotoxicity tests also demonstrated the biocompatibility of treated AZ31 specimens, showing that a physiological *in vitro* growth occurred in a 7 day interval.

## Appendix A

The growth of a barrier anodic film can be described according to the Faraday’s law (see eq 2) that relates the anodic growth rate d*V*/d*t* with the current density *i*, the electric field strength *E*_d_, and the physical parameters of the anodic layers (i.e., density, molecular weight, etc.). From eq 2, *E*_d_ can be easily estimated. Knowing the electric field strength, it is possible to estimate the anodizing ratio, *Ã*, that is the forming factor, according to the following equation^47^

A.1

The knowledge of the anodizing ratio allows us to estimate the barrier anodic layer as the product of *Ã* and the formation voltage. In the case of the anodic growth on AZ31 alloy in glycerol-based and phosphate-containing electrolyte, the voltage useful to estimate the barrier layer thickness is 120 V, that is, until the anodizing voltage increases linearly with time. Considering ρ = 40.3 g cm^–3^, *M* = 40.3 g mol^–1^, and *z* = 2 for MgO and ρ = 2.45 g cm^–3^, *M* = 263 g mol^–1^, and *z* = 6 for Mg_3_(PO_4_)_2_, *E*_d_ = 11.2 MV cm^–1^ and *E*_d_ = 3.5 MV cm^–1^ can be estimated for the growth of pure MgO and pure Mg_3_(PO_4_)_2_, respectively. These electric field strengths lead to *Ã* = 0.9 nm V^–1^ and *Ã* = 2.8 nm V^–1^ for the growth of MgO and pure Mg_3_(PO_4_)_2_ that correspond to 110 and 335 nm as barrier layer thickness, respectively.

## Supporting Information Available

Experimental setup for the HE rate measurement (PDF)
