# Supplementary material for: Tuning of the Mg Alloy AZ31 Anodizing Process for Biodegradable Implants
Source: ACS Appl Mater Interfaces. 2021 Mar 11;13(11):12866–76. doi: 10.1021/acsami.0c22933 (PMC8041254; doi:10.1021/acsami.0c22933)
Supplement: Supplementary file 1 — am0c22933_si_001.pdf [file am0c22933_si_001.pdf]

Supporting Information

Tuning of Mg alloy AZ31 anodizing process for biodegradable implants

Andrea Zaffora[1], Francesco Di Franco[1,], Danilo Virtù[1], Francesco Carfì Pavia[1], Giulio Ghersi[2], Sannakaisa Virtanen[3], Monica Santamaria[1]*

[1]Università degli Studi di Palermo, Dipartimento di Ingegneria, Viale delle Scienze, 90128 Palermo, Italy

[2]Università degli Studi di Palermo, Dipartimento di Scienze e Tecnologie Biologiche, Chimiche e Farmaceutiche (STEBICEF), Viale delle Scienze, 90128 Palermo, Italy

[3]Chair for Surface Science and Corrosion, Department of Materials Science and Engineering, University of Erlangen-Nürnberg, Erlangen 91058, Germany

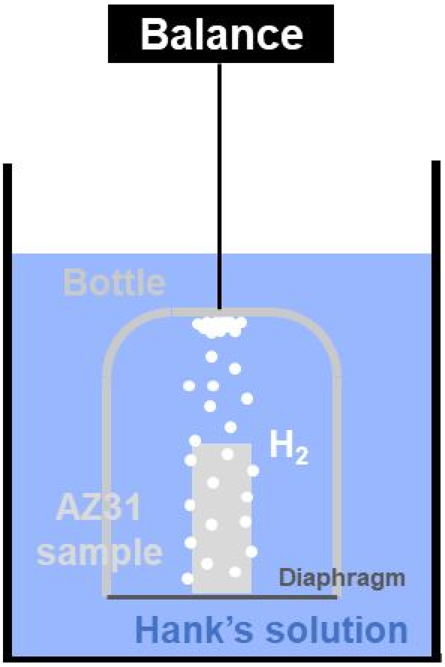

Figure S1. Experimental setup for the hydrogen evolution rate measurement.
